# A combined aging and immune prognostic signature predict prognosis and responsiveness to immunotherapy in melanoma

**DOI:** 10.3389/fphar.2022.943944

**Published:** 2022-08-11

**Authors:** Wenchang Lv, YuanYuan Zhan, Yufang Tan, Yiping Wu, Hongbo Chen

**Affiliations:** Department of Plastic and Cosmetic Surgery, Tongji Hospital, Tongji Medical College, Huazhong University of Science and Technology, Wuhan, China

**Keywords:** aging, immune, immune checkpoint, tumor immune microenvironment, prognosis, melanoma

## Abstract

**Background:** Melanoma is the most lethal, and one of the most aggressive forms of cutaneous malignancies, which poor response to treatment has always puzzled clinicians. As is known to all, aging and immune microenvironment are two crucial factors impacting melanoma biological progress through the tumor microenvironment (TME). However, reliable biomarkers for predicting melanoma prognosis based on aging and immune microenvironment and therapeutic efficacy of immune checkpoints remain to be determined.

**Methods:** The aging-related genes (ARGs) were obtained from the Human Ageing Genomic Resources and immune-related genes (IRGs) were downloaded from the Immunology database as well as Analysis Portal (ImmPort) database. Next, we initially performed LASSO regression and multivariate Cox regression to identify prognostic ARGs and IRGs in the TCGA and GSE65904 datasets, and firstly constructed a novel comprehensive index of aging and immune (CIAI) signature. Finally, *in vitro* molecular biology experiments were performed to assess the regulatory role of CNTFR in melanoma cell lines proliferation and migration, macrophage recruitment, and M2 polarization.

**Results:** This novel CIAI signature consisted of 7 genes, including FOXM1, TP63, ARNTL, KIR2DL4, CCL8, SEMA6A, and CNTFR, in which melanoma patients in the high-CIAI group had shorter OS, DSS, and PFI, indicating CIAI model served as an independent prognostic index. Moreover, we found the CIAI score was potentially correlated with immune scores, estimate score, immune cell infiltration level, tumor microenvironment, immunotherapy effect, and drug sensitivity. Finally, CNTFR might function as oncogenes in melanoma cell lines and the silencing of CNTFR reduced macrophage recruitment and M2 polarization.

**Conclusion:** In this study, we have first presented a novel prognostic CIAI model applied to assess immune checkpoint therapy and the efficacy of conventional chemotherapy agents in melanoma patients. Thus providing a new insight for combating melanoma.

## Introduction

Melanoma is the most lethal, and one of the most aggressive forms of cutaneous malignancies, which all arise from melanocytes at the basal layer of the *epidermis* (Leonardi et al., 2018). Epidemiological studies revealed an upward trend in the incidence of melanoma worldwide (Dimitriou et al., 2021). Melanoma in China is also amongst the malignant tumors that are growing rapidly increasingly prevalent (Yang et al., 2021). The prognosis of melanoma pertains to abundant factors, such as the clinical stage of the patients, and response towards the systematic treatment. Though the 5-years survival rate of melanoma patients has improved since the introduction of targeted therapy, immunotherapy, and multiple treatment schemes the long-term survival of melanoma may still deteriorate suddenly (Zhang and Zhang, 2017; Yan et al., 2021). This is partly attributed to the therapeutic reactivity distinction between individuals, and present condition calls for effective and novel biomarkers for treatment plan selection and prognosis prediction.

Aging and immunity are two crucial factors impacting tumor biological progress through the tumor microenvironment (TME). TME is not only a major regulator of tumor progression but also has profound implications for patient prognosis in the long term. Aging has been deemed a potent inducer of malignancy initiation and progression. Increased age is reported to be linked with poor prognosis of melanoma patients (Page et al., 2012; Tas and Erturk, 2017). Aged non-cancerous cells in TME secret excessive proinflammatory cytokines, growth factors, chemokines, and proteases, and such secretome is named the senescence-associated secretory phenotype (SASP) (Coppe et al., 2010; Meyer et al., 2011). The SASP accelerates age-related cell damage and co-operates with senescent fibroblasts to contribute to a permissive microenvironment for melanoma (Fane and Weeraratna, 2020). Senescent fibroblasts in the stroma have also been reported to increase the invasion of melanoma cells, which is responsible for the deteriorated prognosis in terminal stages of melanoma (Kaur et al., 2016). The role of immune cells in the TME of melanoma is particularly striking, due to, in part, the high tumor mutation burden and the resultant immune cell infiltration in melanoma. Targeted therapy and immune checkpoint therapies have been recognized as first-line treatment strategies for melanoma patients, and prognosis largely depends on whether these treatment strategies can induce sufficient immune response in individual patients (Christiansen et al., 2017). Programmed cell death-1 (PD-1) is an immune checkpoint receptor that promotes melanoma tumor initiation and progression, as well as leading to inhibition of CD8^+^ T cells’ anti-tumor function (Kleffel et al., 2015). Therapeutic antibodies in immune checkpoint therapies targeting programmed cell death-1 (PD-1) have been approved as mature treatment options for melanoma patients (Luke et al., 2017). However, most melanoma patients do not respond to these immunotherapies in a prolonged way (Orloff et al., 2015; Romano and Kwong, 2017). Therefore, outcome prediction or even a customized therapy combination for each patient precisely is preferred nowadays. We attempted to approach this goal in melanoma based on biomarkers associated with aging and immunity in melanoma TME.

In this study, we construct and validated a novel comprehensive index of aging and immune (CIAI) model based on transcriptome sequencing data and clinical information in the TCGA online database. The model demonstrated more stable and accurate performance in predicting the prognosis of melanoma than existing models, which would better guide medical decisions and optimize treatment plans in the clinical practice of melanoma. The detailed flowchart could be seen in Supplementary Figure S1.

## Materials and methods

### Data collection and preprocessing

The latest transcriptome sequencing data and corresponding clinical characteristic information of SKCM patients and other 32 cancer types were obtained from The Cancer Genome Atlas (TCGA) website (https://cancergenome. nih. gov/) (Blum et al., 2018). For TCGA dataset, FPKM value was transformed to TPM value. Notably, the following clinical information such as age, gender, survival status, overall survival (OS), disease-free survival (DFS), progression-free interval (PFI), and TNM staging was also downloaded from the TCGA database and then used for subsequent bioinformatics analysis. To further guarantee the reliability and stability of the analysis, patients without survival information and accurate clinical information were excluded for subsequent evaluation. Additionally, the detailed clinical information and screening criteria of these enrolled SKCM patients were shown in Table 1. A total of 307 human AGs were retrieved from the Human Ageing Genomic Resources and sorted out in Supplementary Table S1. Meanwhile, a total of 6,196 human immune-associated genes (IRGs) were derived from the Immunology Database as well as Analysis Portal (ImmPort) database and sorted out in Supplementary Table S2. To further evaluate the efficacy of immunotherapy in SKCM patients, a urothelial carcinoma dataset containing anti-PD-L1 therapy efficacy was acquired through the R package “IMvigor210CoreBiologies” and applied for external validation (Necchi et al., 2017).

**TABLE 1:** Summary of clinical characteristics of TCGA-SKCM dataset.

Characteristic	TCGA- SKCM data set (*n* = 470)
Vital status, n (%)	
Alive	248 (52.8)
Dead	222 (47.2)
Age, n (%)	
<65	289 (61.5)
≥65	173 (36.8)
Unknow	8 (1.7)
Gender, n (%)	180 (38.3)
Female	290 (61.7)
Male	
WHO-Stage, n (%)	
0	7 (1.5)
Ⅰ	91 (19.4)
Ⅱ	140 (29.8)
Ⅲ	171 (36.3)
Ⅳ	23 (4.9)
Unknow	38 (8.1)
AJCC-T stage, n (%)	
T0	23 (4.9)
T1	42 (8.9)
T2	78 (16.6)
T3	90 (19.1)
T4	153 (32.6)
TX	55 (11.7)
Unknow	29 (6.2)
AJCC-N stage, n (%)	
N0	235 (50.0)
N1	74 (15.7)
N2	49 (10.4)
N3	55 (11.7)
NX	36 (7.7)
Unknow	21 (4.5)
AJCC-M stage, n (%)	
M0	418 (88.9)
M1	24 (5.1)
Unknow	28 (6.0)

### Construction and validation of the comprehensive index of aging and immune

Firstly, based on the SKCM patient’s survival status and OS, the univariate Cox analyses were applied to identify the aging- and immune-related genes in TCGA and GSE65904 datasets, respectively. Secondly, the common prognostic genes in both TCGA and GSE65904 datasets were obtained employing Venn plots. Subsequently, the LASSO regression and multivariate Cox regression analysis were performed to establish a risk signature. Based on the coefficient of risk genes, we calculated the risk score of each SKCM patient, and then all patients were divided into high- and low-risk groups according to the median cutoff value (Chen et al., 2021). The risk score is calculated by the following formula: Risk score = coef gene 1) × exprgene 1) + coef gene 2) × exprgene 2) + … + coef gene (n) × exprgene (n). Meanwhile, the differences in OS, DFS, and PFI between the high- and low-CIAI groups in both the training cohort and testing cohort were evaluated using Kaplan–Meier survival analysis. Finally, the 1-, 3-, and 5-years areas under the ROC curves (AUCs) and Time‐dependent ROC curve analysis were calculated to estimate the predictive value of the CIAI model.

### Immune infiltration analysis and cancer-immunity cycle

The fractions of 22 immune cell types in each SKCM patient were assessed using Estimating Relative Subsets of RNA Transcripts (CIBERSORT) analysis with the “CIBERSORT” R package (Chen et al., 2018). Briefly, the different immune cell subtypes of B-cell, T-cell, natural killer cell, plasma cell, and myeloid cell types were distinguished using the leukocyte gene signature, termed LM22. Furthermore, an increasing number of evidence have demonstrated that the Cancer-Immunity Cycle played an indispensable role in the elimination of cancer, which maintained the delicate balance between the recognition of nonself and the prevention of autoimmunity. To effectively killing of cancer cells, the cancer-immunity cycle must be executed and expand iteratively in the anticancer immune response. The process mainly consists of the following seven steps: 1) cancer antigen release, 2) cancer antigen presentation, 3) initiation and activation, 4) T-cell transport to the tumor, 5) T cells penetration into the tumor, 6) T-cell recognition of cancer cells, and 7) T cell killing of cancer cells.

### Immunotherapy/chemotherapy sensitivity prediction

Encouragingly, immune checkpoint inhibitors have been approved as routine drugs for the treatment of melanoma with remarkable success. Thus, we calculated the tumor immune dysfunction and exclusion (TIDE) score using an online website (http://tide.dfci.harvard.edu/), and then assessed the potential response to immune checkpoint inhibitor (ICI) (Wang et al., 2020). Numerous studies have confirmed that the immunophenoscore was an admirable predictor of response to anti-cytotoxic T lymphocyte antigen-4 (CTLA-4) and anti-programmed cell death protein 1 (anti-PD-1) antibodies. Thus, to further elaborate on the immunophenotypes and tumor escape mechanisms, we also quantitatived the immunophenoscore (IPS) of each SKCM patient in the high- and low-CIAI groups through the Cancer Immunome Atlas (https://tcia.at/). Additionally, the expression levels of three immune checkpoints including PD-1, PD-L1, and CTLA-4 were quantified in the high- and low-CIAI groups.

### Cell culture and transfection

The melanoma cell lines including A375 and A875 were purchased from American Type Culture Collection (ATCC) and then cultured with 10% serum-containing DMEM (Gibco) in a 37°C incubator supplemented with 5% CO2. The small interfering RNAs (siRNAs) and the negative control were designed and synthesized by Ribo Biotech (Guangzhou, China). The sequences were as follows: 5′-GTC​TTT​TCC​TCT​CAA​GTT​CTT​TC-3′ for CNTFR-specific siRNA. Afterward, according to the manufacturer’s instructions, siRNAs were transfected into cells by Lipofectamine 3,000 Transfection Reagent (Invitrogen, United States). After transfection, cells were collected and the silencing efficiency was detected by RT-qPCR analysis.

### CCK8 assay

In this study, the CCK8 assay was performed to evaluate the effect of gene silencing on cell proliferation. Briefly, cells were cultured in a 96-well plate at a density of 3 × 10^3^/well and then transfected with si-NC or si-CNTFR. After transfection for 0, 24, 48, or 72 h, 10 µl CCK-8 reagent (BioTek Instruments, United States) was directly added to each well of a 96-well plate and then incubated for an additional 2 h in a dark environment. Finally, the cell proliferation was measured by an optical density (OD) value at 450 nm through a microplate reader (BioTek Instruments, United States) (Krishan and Hamelik, 2010).

### Wounding healing and transwell migration assays

After transfection with si-NC or si-CNTFR, melanoma cell lines were seeded into a 6-well plate and then scratched with a 200 μl pipette tip when the degree of cell fusion approaches 90%. After incubation with a serum-free medium for 24 h, the width of the wounds was examined under a microscope.

Moreover, the migratory ability of cells was assessed by transwell migration assay using the 24-well transwell migration chambers (8-mm pore size; Corning, NY, United States). In short, cells with a density of 5×10^4^/ml were resuspended in 200-ml serum-free DMEM and then plated into the inner chambers (Omar Zaki et al., 2019). Similarly, a 500 µl DMEM medium containing 20% serum was added to the bottom chambers as an attractant. After 24 h of incubation, cells on the transparent membrane were washed with PBS, fixed with 4% paraformaldehyde for 30 min, and counterstained with 0.1% crystal violet. Finally, the number of migrated cells was calculated by ImageJ software.

### Statistical analysis

Statistical evaluations of molecular biology experiments and bioinformatics analyses were carried out using GraphPad Prism 8 and R 3.6.3 (https://www.r-project.org/), respectively. The overall survival curves, DFS, and PFI were undertaken with Kaplan–Meier survival analysis. As appropriate, the significance between the high- and low-CIAI groups was calculated using the 2-tailed Student’s t-test. In this study, all molecular biology experiments were performed in triplicate, and values were expressed as mean ± standard deviation (SD). For all the data, statistical significance was considered statistically significant as a level of *p* less than 0.05.

## Results

### Construction of comprehensive index of aging and immune in melanoma

**FIGURE 1:**
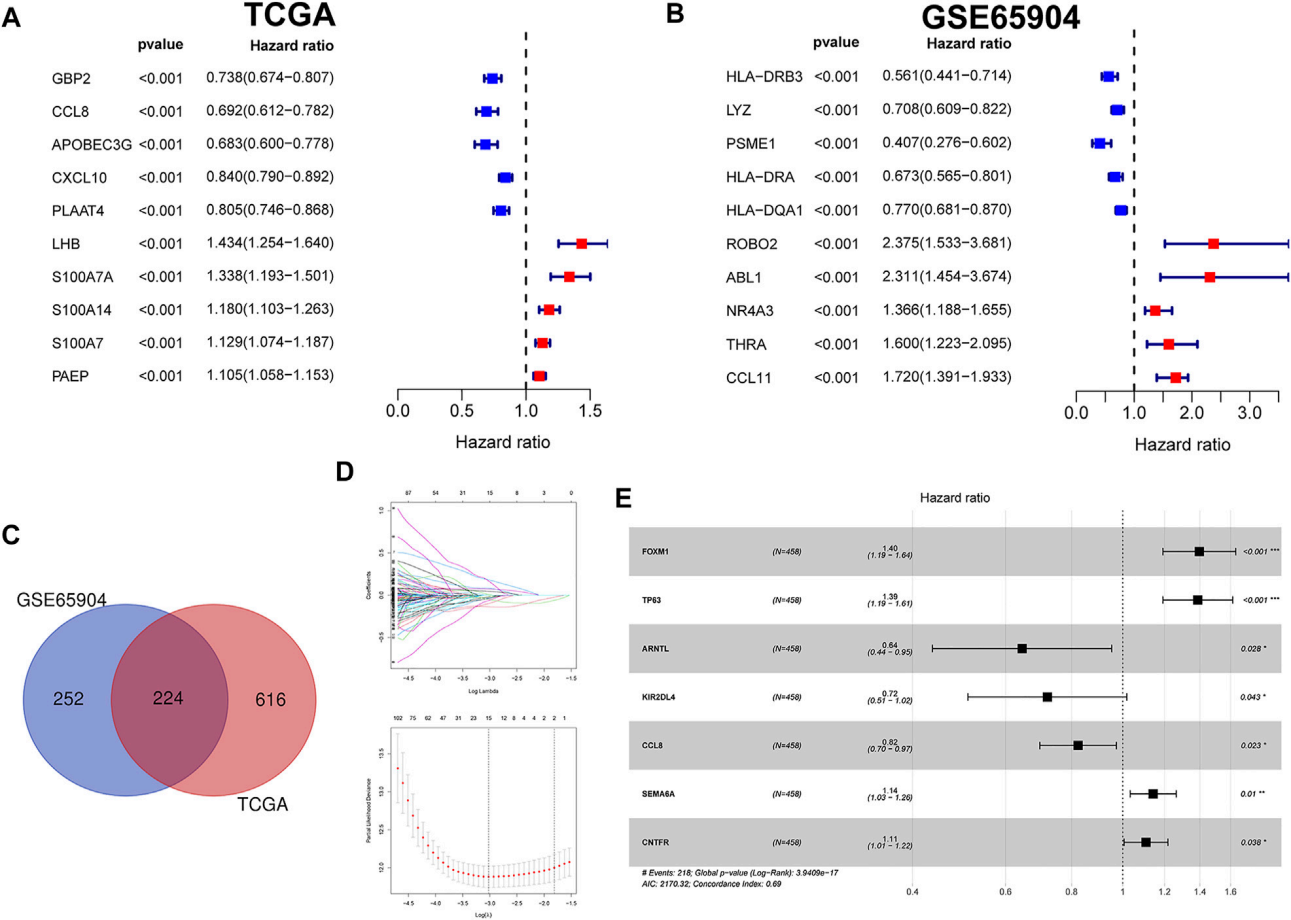
Identification and verification of prognostic aging and immune genes in melanoma. **(A)** The univariate Cox regression analysis revealed hazard ratios of the top 10 genes correlated with Melanoma prognosis in the TCGA **(A)** and GSE65904 datasets **(B)**, respectively. **(C)** Venn diagram showing a total of 224 common prognostic ARGs and IRGs were selected between the TCGA dataset and the GSE65904 dataset. The LASSO regression analysis **(D)** and stepwise multivariate Cox regression analysis **(E)** were employed to narrow the most relevant prognostic genes.

In this study, the univariate Cox regression analysis was carried out to screen out genes significantly correlated with Melanoma prognosis in the TCGA and GSE65904 datasets, respectively. In the TCGA dataset, a total of 840 genes were significantly correlated with overall survival in patients with melanoma and then the top ten genes with the lowest *p* values were visualized (Figure 1A). Likewise, in the GSE65904 dataset, a total of 476 genes were significantly correlated with overall survival in patients with melanoma and then the top ten genes with the lowest *p* values were visualized (Figure 1B). Then, the intersection of CIAI genes between the TCGA dataset and the GSE65904 dataset was determined, and a total of 224 common prognostic ARGs and IRGs were selected and visualized on a Venn diagram (Figure 1C). Considering the exceedingly large number of genes is not conducive to clinical promotion, the LASSO regression analysis and stepwise multivariate Cox regression analysis were employed to narrow the number of genes and construct a prognostic gene model (Figures 1D,E). Based on the optimal lambda value, the CIAI model was constructed and contained 7 genes, including FOXM1, TP63, ARNTL, KIR2DL4, CCL8, SEMA6A, and CNTFR (Figure 1E). The model formula was as follows: The risk score = FOXM1 × (0.0065) + TP63 × (-0.0038) + ARNTL × (-0.0154) + KIR2DL4 × (0.0177) + CCL8 × (-0.0237) + SEMA6A × (0.0014) + CNTFR × (-0.0071). Furthermore, the Kaplan-Meier analysis revealed that among those 10 CIAI genes, the high expression of FOXM1, TP63, SEMA6A, and CNTF predicted poor survival prognosis, while ARNTL, KIR2DL4, and CCL8 predicted the opposite conclusion (Supplementary Figure S2).

### Establishment and validation of comprehensive index of aging and immune model in the cancer Genome Atlas dataset

**FIGURE 2:**
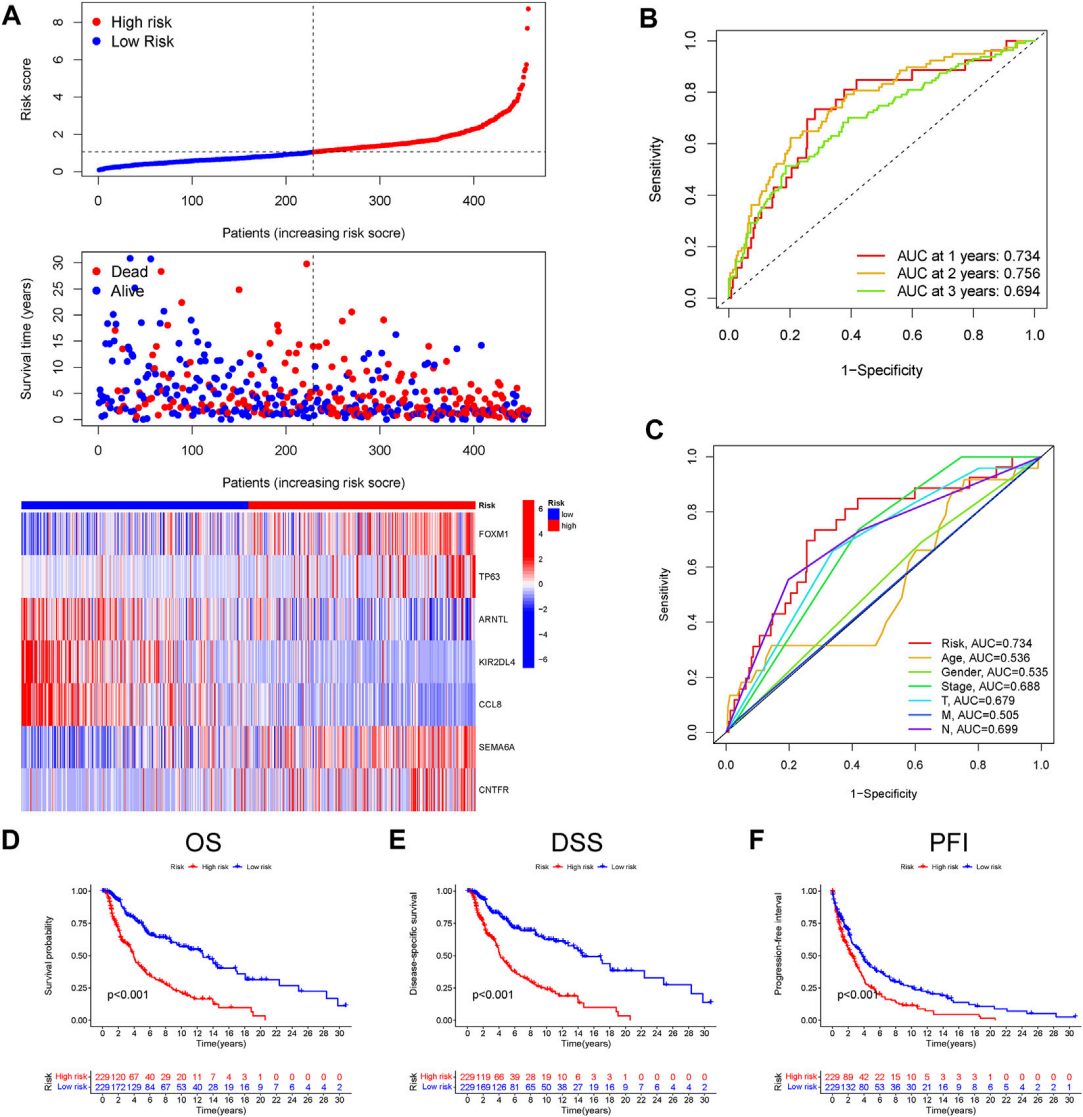
Prognostic analysis of CIAI in the TCGA dataset. **(A)**The distribution of risk scores, survival status, and CIAI expression patterns of each melanoma patient in the TCGA dataset was visualized. **(B)** The ROC curve analyses showing the AUC values for the 1-, 2-, and 3-years survival rates were 0.734, 0.756, and 0.694, respectively. **(C)** The ROC curve analyses showing the prognostic accuracy of risk score, age, stage, and TNM staging in the TCGA dataset. The Kaplan-Meier analysis of the OS **(D)**, DSS **(E)**, and PFI **(F)** in the high-versus low- CIAI group.

According to the above-calculated formula, the risk score of each melanoma patient in the TCGA cohort was calculated and distribution was visualized in Figure 2A. Simultaneously, patients were divided into either high- or low-risk CIAI groups with the median threshold of C IAI score. Notably, the ROC curve analyses revealed that the AUC values for the 1-, 2-, and 3-years survival rates were 0.734, 0.756, and 0.694, respectively, indicating that the model possesses predominant predictability (Figure 2B). Additionally, the AUC of the risk score pointing to 0.734 was superior to those of other independent clinicopathological variables (Figure 2C). Likewise, results from the Kaplan-Meier survival analyses exhibited that patients in the high-CIAI group had shorter OS (*p* < 0.001; Figure 2D), DSS (*p* < 0.001; Figure 2E), and PFI (*p* < 0.001; Figure 2F) compared to the low-CIAI group.

### Establishment and validation of comprehensive index of aging and immune model in the GSE65904 dataset

**FIGURE 3:**
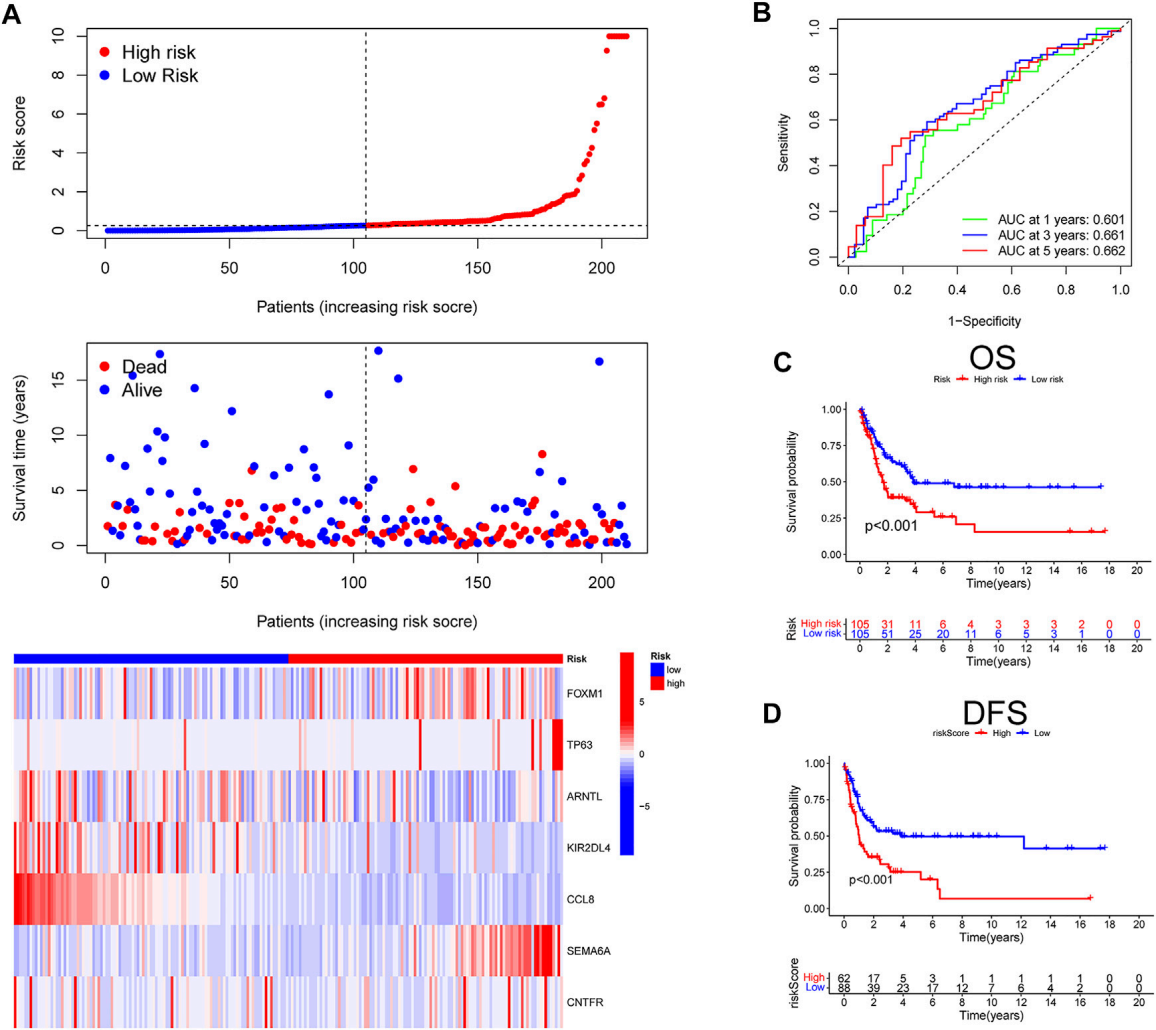
Prognostic analysis of CIAI in the GSE65904 dataset. **(A)**The distribution of risk scores, survival status, and CIAI expression patterns of each melanoma patient in the GSE65904 dataset was visualized. **(B)** The ROC curve analyses showing the AUC values for the 1-, 2-, and 3-years survival rates were 0.601, 0.661, and 0.662, respectively. The Kaplan-Meier analysis of the OS **(C)** and DFS **(D)** in the high-versus low- CIAI group.

Next, to validate the stability and versatility of the CIAI model, the CIAI score was calculated in the GSE65904 dataset, termed as a testing cohort. The CIAI score distribution and CIAI gene expression pattern in the GSE65904 dataset were visualized in Figure 3A. The ROC curve analyses revealed that the AUC values in the testing cohort for the 1-, 2-, and 3-years survival rates were 0.601, 0.661, and 0.662, respectively (Figure 3B). Moreover, results from the Kaplan-Meier survival analyses exhibited that patients in the high-CIAI group also had shorter OS (*p* < 0.001; Figure 3C) and DFS (*p* < 0.001; Figure 3D) compared to the low-CIAI group.

### Development of a nomogram of melanoma patients

**FIGURE 4:**
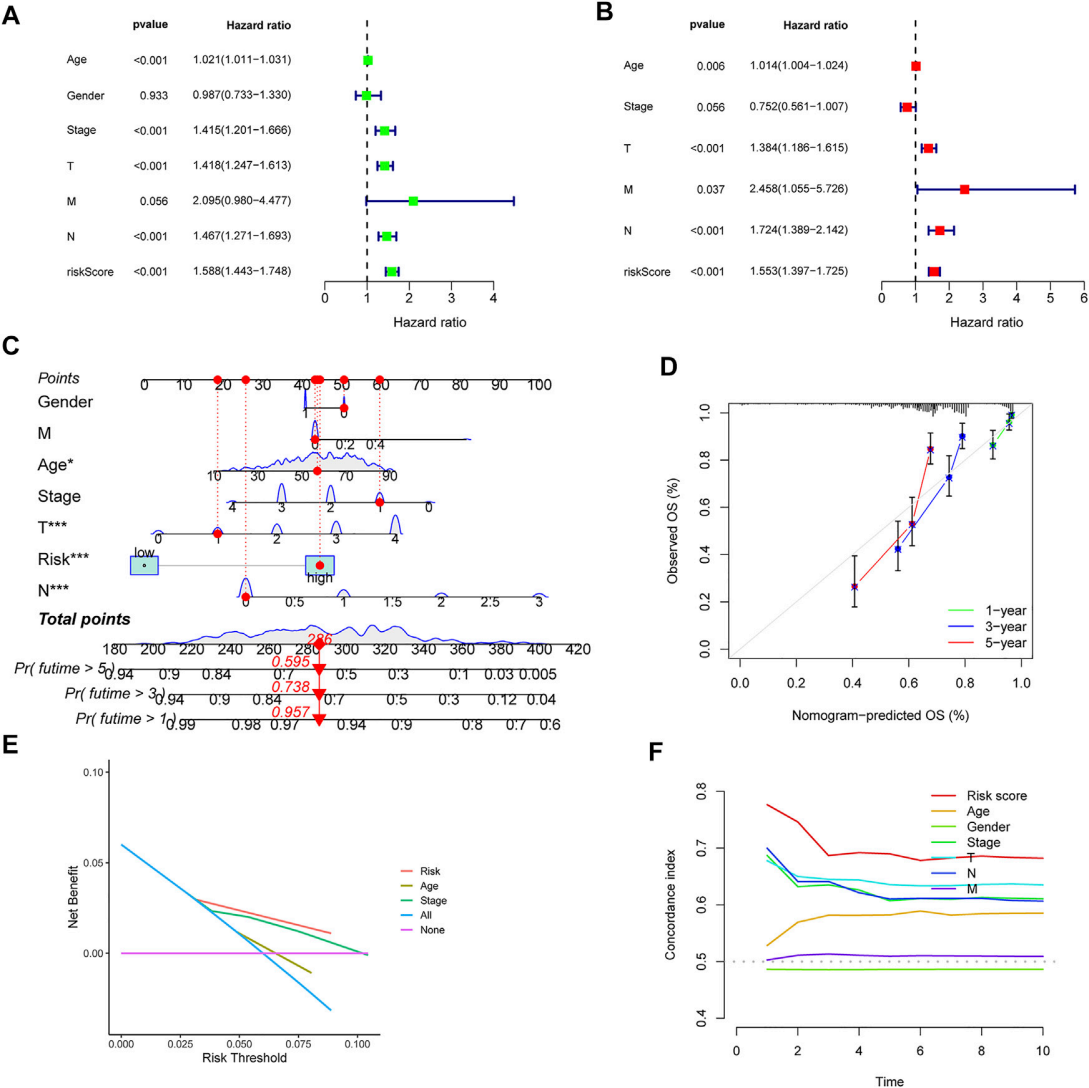
Univariate and multivariate cox analyses of CIAI and the prognostic accuracy of risk score, age, stage, and TNM staging were compared. The univariate **(A)** and multivariate **(B)** Cox regression analyses of the risk score and other clinical feature prognostic values. **(C)** Based on all independent factors nomogram was constructed to predict the 1-, 3-, and 5-years OS of melanoma patients. **(D)** The calibration curve for the prediction and observed 1-, 3-, and 5-years OS. **(E)** DCA of the nomogram model. **(F)** C-index of the nomogram model.

Firstly, the univariate and multivariate Cox regression analyses were employed to confirm the independent prognostic value of the CIAI score compared with other clinicopathological parameters. Notably, the Univariate COX analysis demonstrated that CIAI (HR = 1.588, *p* < 0.001), age (HR = 1.021, *p* < 0.001), clinical-stage (HR = 1.415, *p* < 0.001), T stage (HR = 1.418, *p* < 0.001), and N stage (HR = 1.467, *p* < 0.001) were all markedly relevant to the prognosis of melanoma patients (Figure 4A). Besides, the Multivariate COX analysis also demonstrated that CIAI (HR = 1.553, *p* < 0.001), age (HR = 1.014, *p* = 0.006), T stage (HR = 1.384, *p* < 0.001), and N stage (HR = 1.724, *p* < 0.001) were all markedly relevant to the prognosis of melanoma patients (Figure 4B). Taken together, results from the Cox regression analyses indicated that the CIAI score stood for an independent risk factor for prognosis in melanoma patients. Subsequently, for better clinical practice, based on all independent factors, a nomogram was constructed to predict the 1-, 3-, and 5-years OS of melanoma patients (Figure 4C). Meanwhile, the calibration curve analysis was performed to detect the reliability and accuracy of the nomogram. The result showed that the forecasting curve of 1-, 3-, and 5-years OS was ideally consistent with the actual observed OS (Figure 4D). Thus, this nomogram could be superiorly applied in clinical practice to predict the survival rate of melanoma patients. Next, the decision curve analysis (DCA) showing the nomogram can better predict OS than Traditional single clinicopathological features (Figure 4E). Finally, the concordance index (C-index) was calculated through the “RMS” package in R. The result proved that the AUC values of risk core were the highest than that of age, gender, stage, and stages T, N, and M (Figure 4F).

### Comparison of the comprehensive index of aging and immune model with others

**FIGURE 5:**
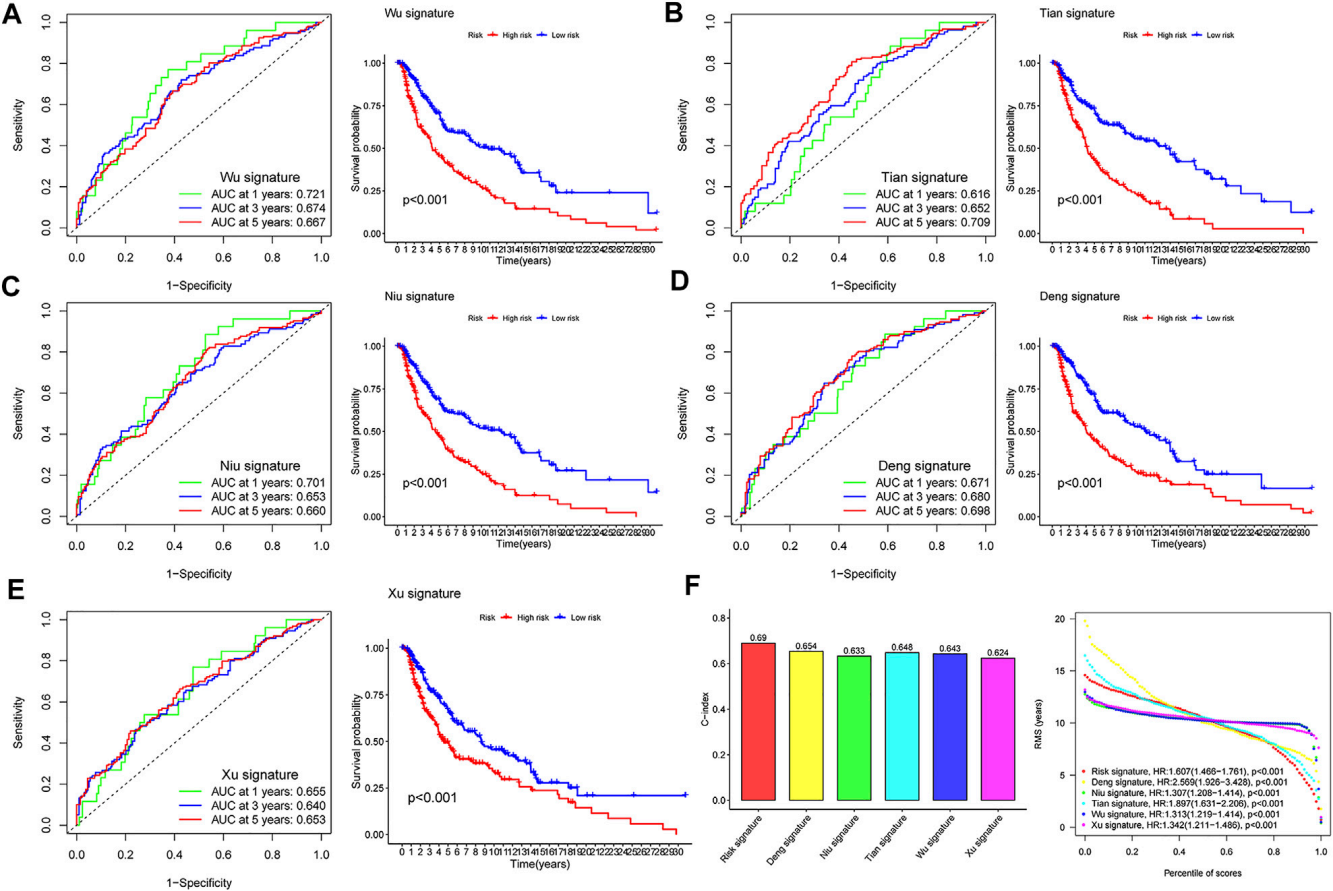
Comparison of risk models. **(A–E)** The ROC curve and KM curve of the high-versus low-risk group in the already published signature of Wu, Tian, Niu, Deng, and Xu signatures. **(F)** C-index comparison and restricted meansurvival (RMS) curves of six prognostic risk models.

After reviewing the published literature on melanoma prediction models, we compared our constructed CIAI model with the following models including the Wu signature, Tian signature, Niu signature, Deng signature, and Xu signature. Firstly, according to the coefficient of risk genes, we calculated the risk score of each patient in the TCGA SKCM dataset. Then, the Kaplan-Meier survival analyses were used to compare the survival status of samples in the high- and low-risk groups in different models. Interestingly, all results showed that patients in the high-risk group were behalf of worse clinical outcomes (*p* < 0.01), and the AUC of these 5 published models at 1, 3, and 5 years were markedly lower than the CIAI model (Figures 5A–E). Besides, the concordance index (C-index) of these models was computerized using the “RMS” package. From the C-index results, it can be seen that the CIAI model had the highest C-index than that of the other four models, indicating improved performance (Figure 5F).

### The correlation of the comprehensive index of aging and immune score with the clinical characteristics in patients with melanoma

**FIGURE 6:**
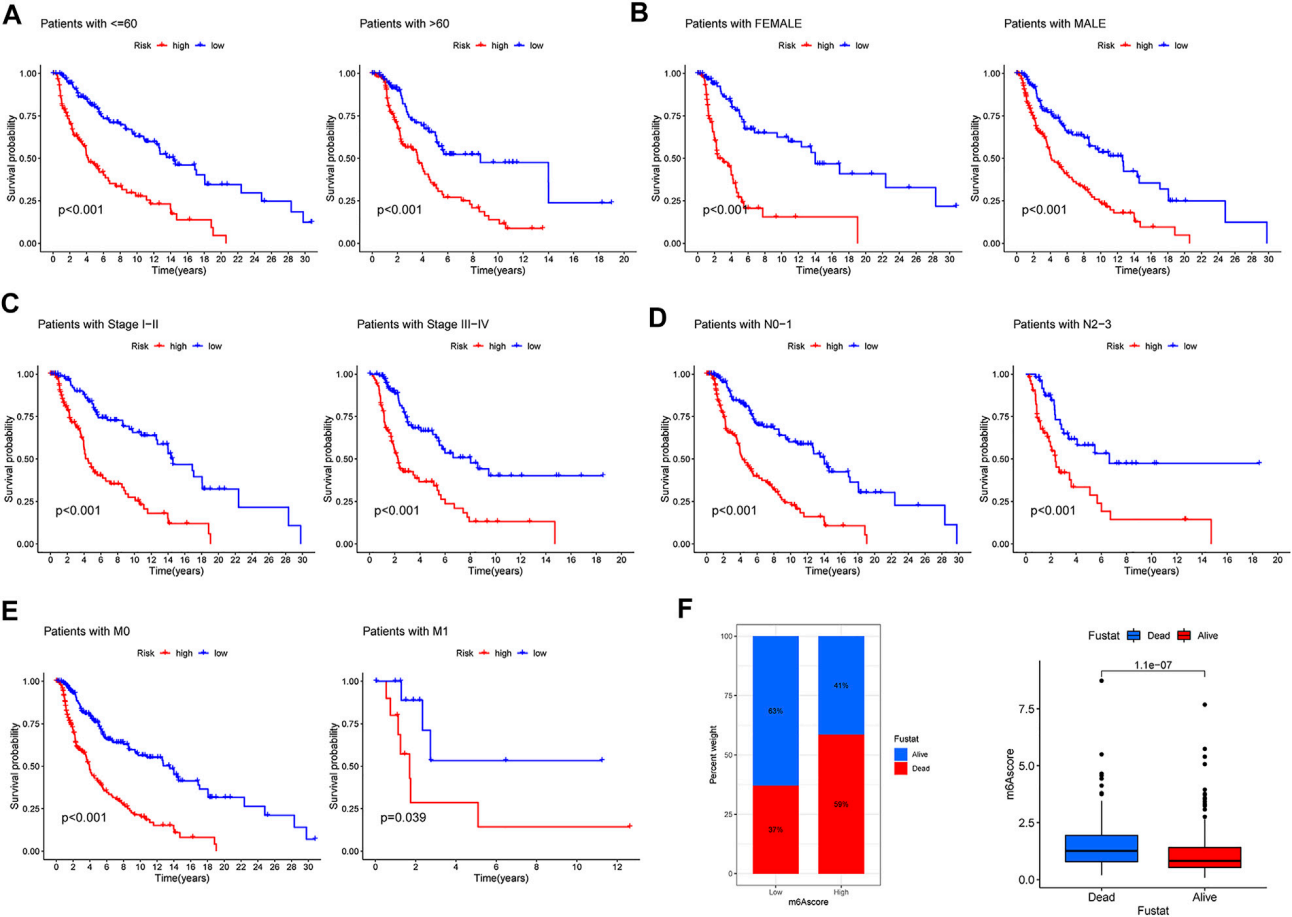
Prognostic significance of CIAI in melanoma patients with different clinical features. **(A)** Age <60 and Age >60. **(B)** Female and Male. **(C)** Stage Ⅰ-II and Stage III-IV. **(D)** N0-1 and N2-3. **(E)** M0 and M1. **(F)** The survival time in the high-CIAI group was observably shorter compared to the low-CIAI group among melanoma patients.

To further measure the clinical application value of CIAI, we carried out a survival analysis of the different clinical characteristics including age, gender, clinical stage, TNM staging, and survival status based on the CIAI score. Interestingly, the CIAI model was able to distinguish between patients with age >65, age <= 65, Male, Female, Stage I + II, Stage III + IV, N0-1, N2-3, M0, and M1, respectively. Among melanoma patients with different clinical characteristics, the survival time in the high-CIAI group was observably shorter compared to the low-CIAI group, demonstrating the accuracy and predictability of CIAI model (Figures 6A–F). Consequently, we can conclude that our model functioned favorably in predicting various clinical signs.

### Characteristics of immune cells infiltration in melanoma

**FIGURE 7:**
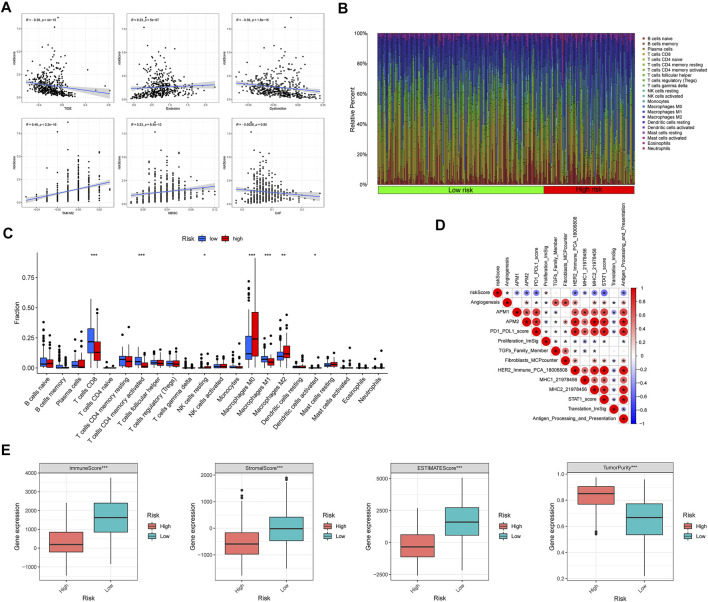
Relationship between CIAI model and immune status. **(A)** Correlation analysis between CIAI model and TIDE, Exclusion, Dysfunction, M2 subtype of tumor-associated macrophage (TAM), myeloid-derived suppressor cell (MDSCs), and tumor-associated fibroblast (CAF). **(B)** The CIBERSORT algorithm showing the infiltration levels of 28 immune cell types were profiled in melanoma samples. **(C)** Correlation between CIAI model and 24 immune cells. **(D)** Spearman’s correlation was performed to analyze the correlation between CIAI model and known gene characteristics. **(E)** RiskScore and ImmuneScore, StromalScore, ESTIMATEScore, and TumorPurity correlation analysis in TCGA dataset.

With a higher TIDE prediction score, there was a greater emphasis on immune escape, indicating that patients who received ICI had a less favorable treatment effect. Our study demonstrated that the CIAI score was negatively corroborated with TIDE, thus the ICI treatment may be more beneficial for low CIAI score patients (Figure 7A). Meanwhile, we also found that the CIAI score was positively correlated with TAM M2, MDSC, and Exclusion levels, but negatively correlated with Dysfunction (Figure 7A). Utilizing the CIBERSORT algorithm, infiltration levels of 28 immune cell types were profiled in melanoma samples (Figure 7B). With the exclusion of CD8 T cells (*p* < 0.001), activated memory CD4 T cells (*p* < 0.001), and Macrophages M1 (*p* < 0.001) were all notably up-regulated in the low-CIAI group (Figure 7C). On the contrary, the infiltration levels of resting NK cells (*p* < 0.05), Macrophages M0 (*p* < 0.001), Macrophages M2 (*p* < 0.01), and Dendritic cells activated (*p* < 0.05) were all notably up-regulated in the high-CIAI group (Figure 7C). The correlation between the CIAI score and important biological pathways was further explored, and it can be seen that the CIAI score was positively correlated with Proliferation_ImSig but negatively correlated with Angiogenesis, APM1, APM2, PD1_PDL1_score, HER2_Immune_PCA, MHC1, MHC2, STAT1_score, and Antigen_Processing_and_Presentation (Figure 7D). In addition to calculating immune and matrix scores through the ESTIMATE algorithm, and observed further that ImmuneScore, StromalScore, and ESTIMATEScore were the higher in the low CIAI score group, with the high CIAI score group having the higher TumorPurity (Figure 7E).

### comprehensive index of aging and immune score predicts the response to immunotherapy in melanoma

**FIGURE 8:**
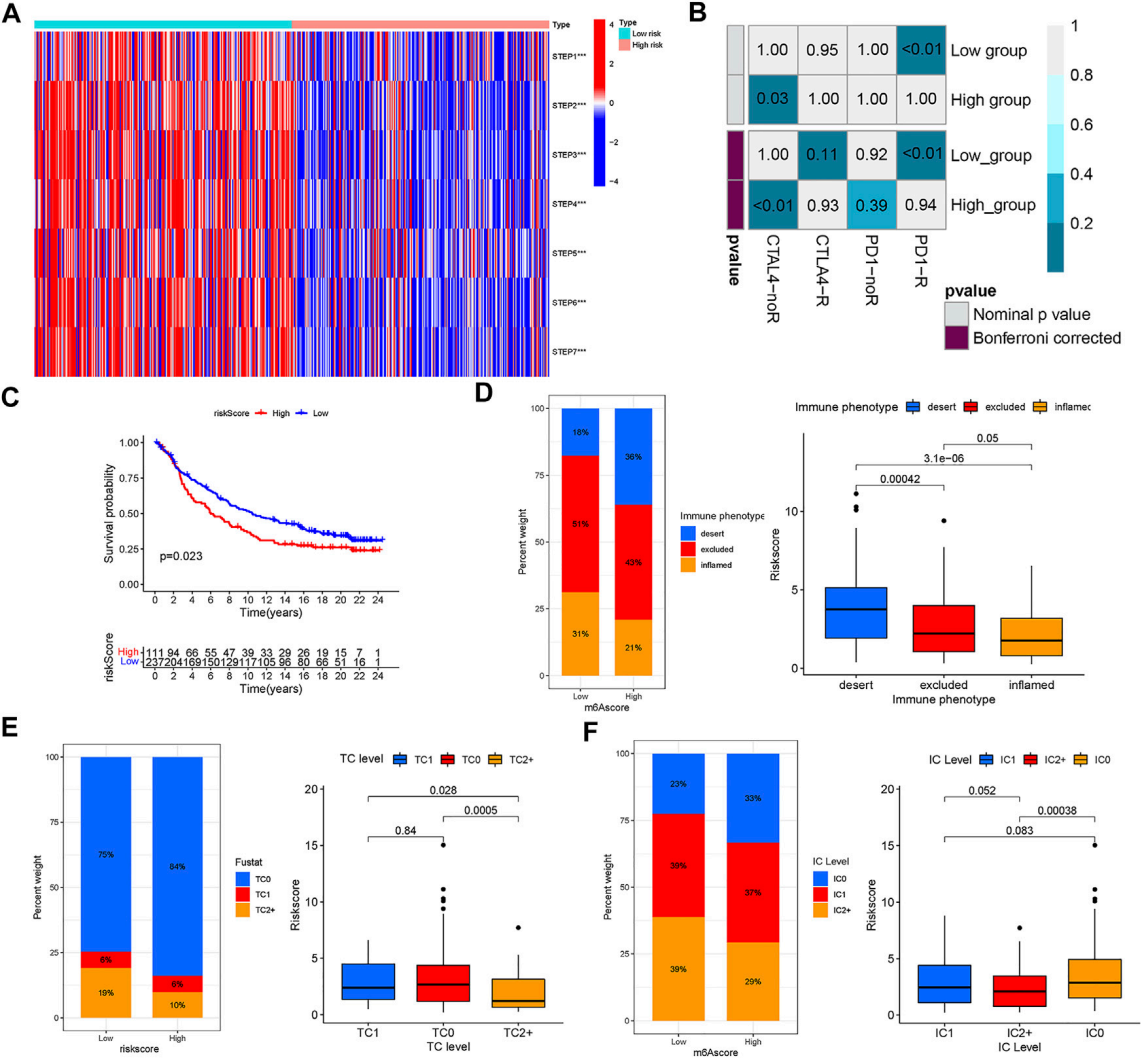
The riskscore predicts the responsiveness to immunotherapy. **(A)** The scores for seven steps of the Cancer-Immunity Cycle were calculated through the ssGSEA algorithm. **(B)** The SubMAP algorithm revealing the possibility of anti-PD1 and anti-CTLA4 response immunotherapy in the high-versus low- CIAI group. **(C)** Kaplan-Meier curve showed that patients with low CIAI score had a better prognosis following treatment with immunotherapy in the IMvigor210 cohort. **(D)** The CIAI score was tested at three immunophenotype levels using the Kruskal-Wallis test. The result of the Kruskal-Wallis test revealed the CIAI score of PD-L1 expression on tumor cells **(E)** and PD-L1 expression on immune cells **(F)**.

With the help of the ssGSEA algorithm, we calculated the scores for seven steps of the Cancer-Immunity Cycle using the essential regulatory genes. With this analysis, we found that the low CIAI score group had a more aggressive tumor immune response (Figure 8A). The submap algorithm was applied to forecast the response to anti-PD1 and anti-CTLA4 immunotherapy of high and low CIAI score groups. Interestingly, there was evidence that the low CIAI score group might benefit more from anti-PD1 treatments (Bonferroni corrected *p* < 0.01: Figure 8B). In the IMvigor210 cohort, patients were divided into two groups based on their CIAI score. As shown in Figure 8C, patients with low CIAI score had a better prognosis following treatment with immunotherapy.

Human solid tumors are characterized by one of three distinct immunological phenotypes: immune inflamed, immune excluded, or immune desert, as well as PD-L1 staining. In addition, the average CD8^+^ Teff signal signature is lowest in deserts, intermediate in excluded tumors, and highest in inflamed tumors, and closely related to response in inflamed tumors (Mariathasan et al., 2018). There is evidence, largely from melanoma, that inflamed tumors respond best to checkpoint blockade. In Figure 8D, we also found a correlation between a low CIAI score and inflamed immunophenotype, which supported the previous conclusion. Tumor cell (TC) 1/2/3 or immune cell (IC) 1/2/3 were defined as the proportion of tumor cells or tumor-infiltrating immune cells expressing positive PD-L1. The result revealed that there was a negative correlation between the CIAI score and PD-L1 protein expression on tumor cells and immune cells (Figures 8E,F). Based on these results, the CIAI score was capable of predicting the outcome of anti-PD-L1 treatment.

### The comprehensive index of aging and immune score and patients’ response to immune checkpoint inhibitor treatment

**FIGURE 9:**
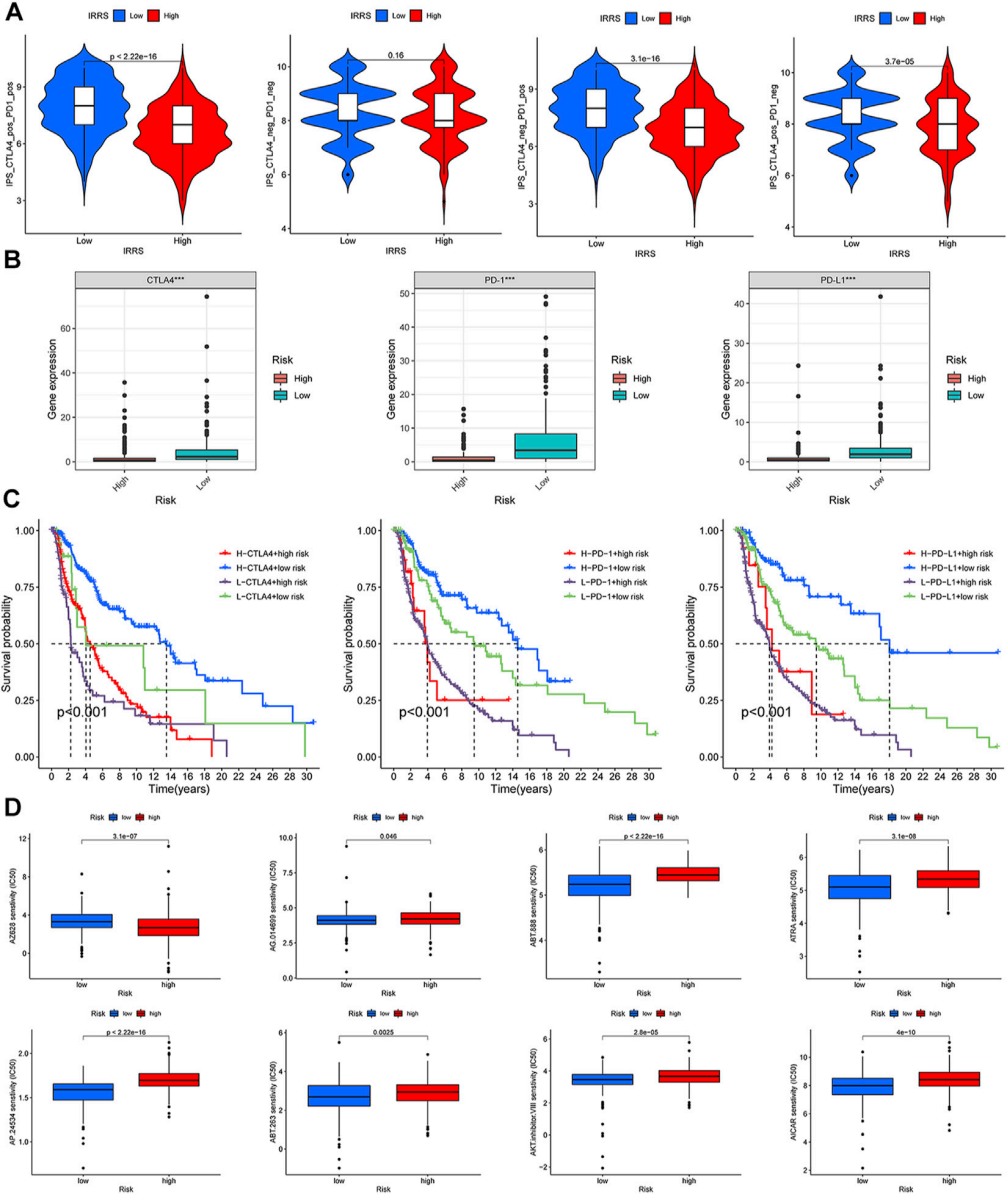
Relationship between CIAI model and immune checkpoint and chemotherapeutic drug sensitivity. **(A)** Four subtypes of IPS values (ips_CTLA-4_pos_PD-1_pos, ips_CTLA-4_neg_PD-1_pos, ips_CTLA-4_pos_PD-1_neg, and ips_CTLA-4_neg_PD-1_neg). **(B)** The expression of PD1, PD-L1, and CTLA-4 in the high-versus low- CIAI group. **(C)** Kaplan-Meier curve analysis of patients with different combinations of CIAI score and immune checkpoint in TCGA cohort. **(D)** Drug sensitivity of ABT.263, ABT.888, AP.24534, AKT. inhibitor.VIII, AICAR, AG.014699, and ATRA.

According to a machine learning-based scoring scheme, the immunophenoscore (IPS) was a superior predictor of response to anti-cytotoxic T lymphocyte antigen-4 (CTLA-4) and anti-programmed cell death protein 1 (anti-PD-1) antibodies (10.1016/j.celrep.2016.12.019). The IPS scores of four various subtypes (CTLA4_neg_PD1_neg, CTLA4_pos_PD1_neg, CTLA4_neg_PD1_pos, and CTLA4_pos_PD1_pos) were applied to predict the response of the melanoma patients to anti-CTLA4 and anti-PD1 treatment. As shown in Figure 9A, patients with low CIAI score had increased response rates to anti-PD1 or anti-CTLA4, and the same pattern was apparent in combination treatment with anti-PD1 and anti-CTLA4. Specifically, the levels of CTLA-4, PD-1 and PD-L1 mRNA were observably raised in the low CIAI score group in comparison with the high CIAI score group (Figure 9B).

In line with this phenomenon, the high expression of immune checkpoint genes predicted a poor prognosis. Combining the CIAI score with immune checkpoint genes can clearly enable better patient stratification. The survival rate of patients with a high CTLA4 and low CIAI score was higher than that of patients with low CTLA4 and high CIAI score (*p* < 0.001). As well, there were similar survival patterns in the CIAI score and PD-1 or PD-L1 (*p* < 0.001; Figure 9C).

We analyzed the IC50 of eight drugs to determine whether the CIAI score correlated with the responsiveness of chemotherapy and target therapy. The estimated IC50 values of ABT.263, ABT.888, AP.24534, AKT. inhibitor.VIII, AICAR, AG.014699, and ATRA in the high CIAI score group were higher than those in the low CIAI score group, which indicated that high CIAI score patients were more resistant to drugs (Figure 9D). Similarly, patients in high-risk group were associated with increased sensitivity to Gefitinib, Vinblastine, and Sunitinib relative to low-risk patients (*p* < 0.05). Inversely, there was a higher sensitivity to AZ628 in patients with high CIAI score groups than in low CIAI score groups.

### Extending the comprehensive index of aging and immune model of melanoma to pan-cancer

**FIGURE 10:**
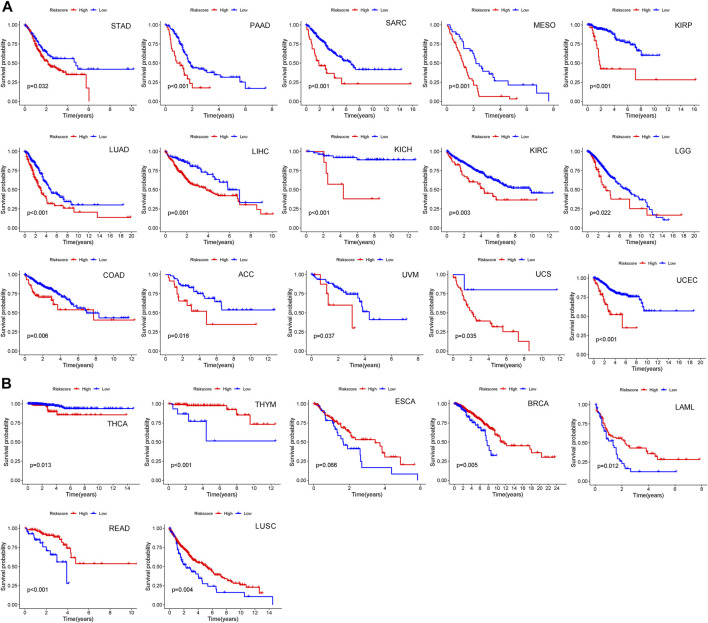
The predictive effect of CIAI model in pan-cancer patients. **(A,B)** Among 32 cancer types, we found CIAI model was markedly relevant to overall survival in 23 cancer types. A high score might predict both worse **(A)** and better **(B)** overall survival.

To further determine whether the CIAI model of melanoma holds true for other cancers in the TCGA dataset, the risk scores of 32 other cancers (except melanoma) were calculated based on the CIAI model formula. Next, we applied the best median as the optimal cut point to divide patients into high- or low-risk groups per cancer type. The result from Kaplan–Meier survival curve analysis revealed that CIAI model was markedly relevant to overall survival in 23 cancer types among 32 cancer types. In STAD, PAAD, SARC, MESO, KIBP, LUAD, LIHC, KICH, KIRC, LGG, COAD, ACC, UVM, UCS, UCEC, and THCA, the risk score obtained from CIAI model formula was proved to be an adverse prognostic factor (Figure 10A), while the risk score predicted favorable survival in THYM, ESCA, BRCA, LAML, and LUSC (Figure 10B). In conclusion, these results indicated that the CIAI model of melanoma might have general prognostic significance for pan-cancer analysis.

### The regulation of CNTFR on the proliferation and migration in melanoma cell lines

**FIGURE 11:**
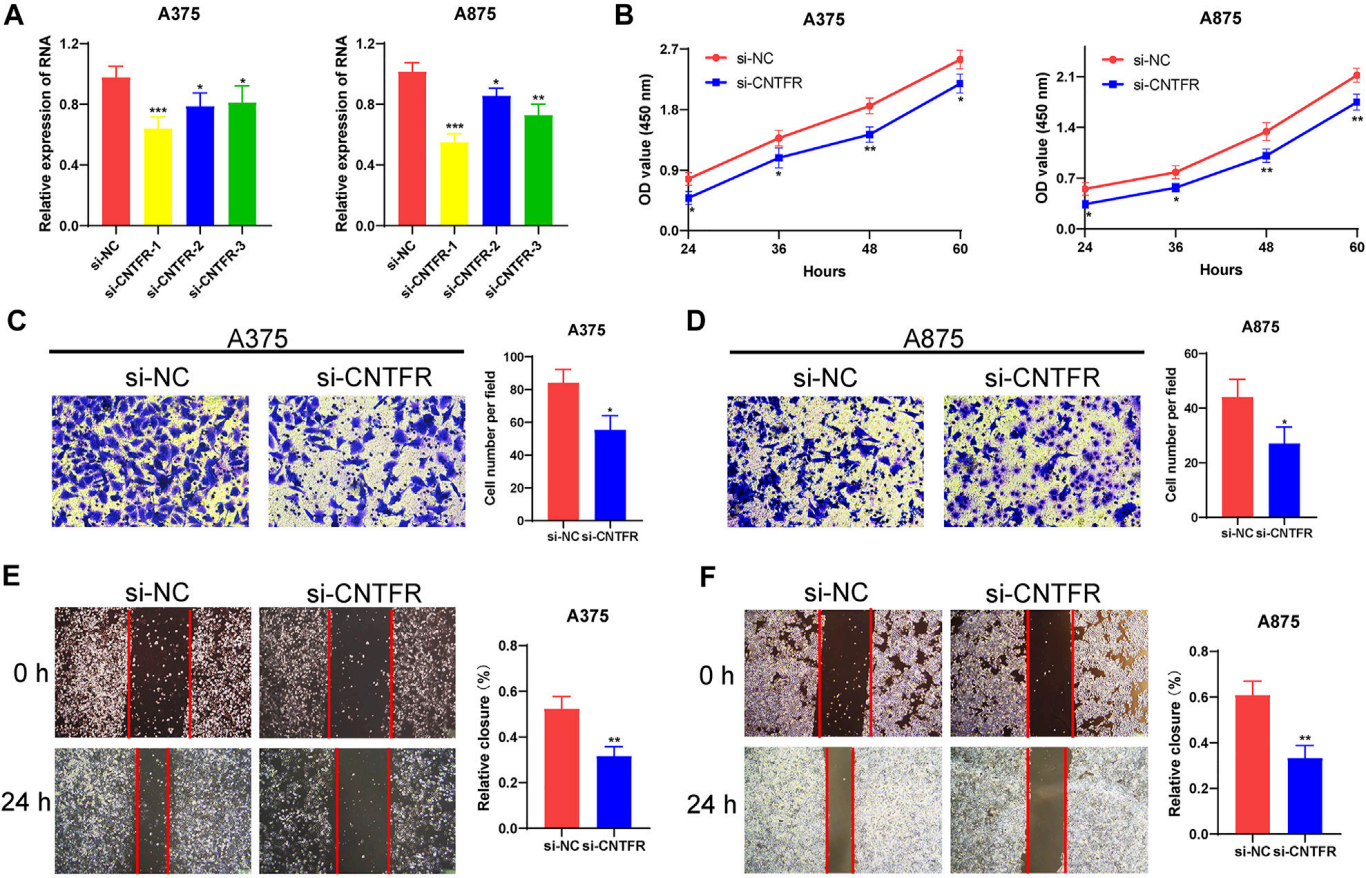
CNTFR regulated the proliferation and migration of melanoma cell lines. **(A)** The expression level of CNTFR in A375 and A875 after transfection with si-NC or si-CNTFR. **(B)** CCK-8 assays were applied to detect the proliferation ability of A375 and A875 after silencing CNTFR. **(C,D)** Transwell migration assays (magnification, ×200) and **(E,F)** wound healing assays (magnification, ×20) were performed to evaluate the migration ability in A375 and A875 cells.

To further detect the regulatory role of CNTFR in melanoma cell lines, we examined whether the silencing of CNTFR played an indispensable role in the proliferation and migration of A375 and A875 cells. After transfection of si-NC and si-CNTFR, respectively, si-CNTFR could observably inhibit the expression of CNTFR in A375 and A875 cells compared with the si-NC group (Figure 11A). The CCK-8 analysis showed that the depletion of CNTFR markedly suppressed the proliferation in both A375 and A875 cells (Figure 11B). Subsequently, we further extended the analysis on cell migration using the transwell migration assay and wound healing assay in A375 and A875 cells. After transfection with si-CNTFR, both A375 and A875 cells demonstrated reduced migration (Figures 11C–F).

### The regulation of CNTFR on macrophage recruitment and M2 polarization

**FIGURE 12:**
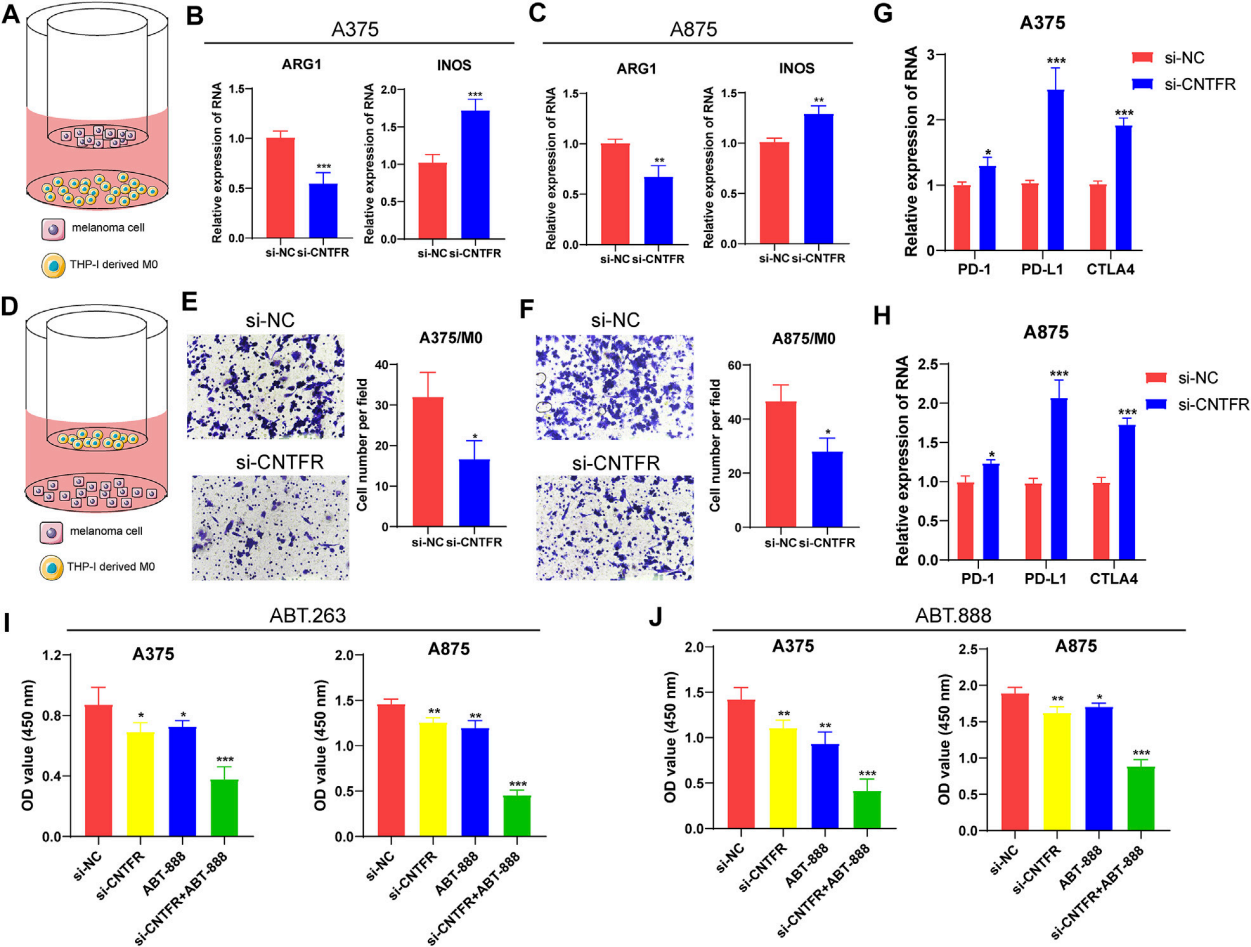
The silencing of CNTFR inhibited macrophage recruitment and M2 polarization. **(A)** Pattern diagram of the THP-1 derived macrophages co-cultured with melanoma cell lines. The expression of ARG1 and INOS was evaluated in THP-1-derived macrophages co-cultured with A375 **(B)** and A875 **(C)**. **(D)** Schematic diagram of macrophage migration. The migration ability of macrophages was significantly reduced when co-cultured with PURPL silenced A375 **(E)** and A875 **(F)** cells (magnification, ×200). The downregulation of CNTFR decreased the expression level of immune checkpoints containing PD-1, PD-L1, and CTLA4 in A375 **(G)** and A875 **(H)** cells. CCK-8 assays were performed to detect the proliferation ability of both the A375 and A875 cells upon exposure to the chemotherapy drugs of ABT-263 **(I)** and ABT-888 **(J)**.

To further verify the immune infiltration analysis in bioinformatics, biological experiments were performed on the regulatory role of CNTFR on macrophage recruitment and M2 polarization. The co-culture system was applied to investigate the polarization of macrophages (Figure 12A). Thp-1 cells, as a human mononuclear cell line, were usually induced by phorbol-12-myristate-13-acetate (PMA) into M0 macrophages. After co-culturing CNTFR silenced melanoma cell lines (A375 and A875) with PMA-induced M0 macrophages for 48 h, the expression levels of macrophage polarization markers were evaluated. It was found that the CNTFR silenced in melanoma cell lines increased the mRNA levels of INOS in THP-1 derived macrophages, whereas inhibited ARG1 expression (Figures 12B,C). Therefore, these results indicating the down-regulation of CNTFR markedly suppressed M2 polarization in TME. The chemotaxis assay was applied to examine the impact of CNTFR on the recruitment of macrophages (Figure 12D). As expected, reducing the expression of CNTFR in A375 and A875 cells could significantly suppress the migration capability of co-cultured macrophages (Figures 12E,F). Taken together, the expression of CNTFR in melanoma cell lines proved a pivotal role in macrophage recruitment in TME. Interestingly, the downregulation of CNTFR decreased the expression level of immune checkpoints containing PD-1, PD-L1, and CTLA4 in A375 and A875 cells (Figures 12G,H), indicating that patients in the low-risk group responded better to immune checkpoint therapy (Figures 12G,H). Furthermore, to investigate the effect of chemotherapy drugs (ABT-263 and ABT-888) and si-CNTFR combined therapy on the proliferation of melanoma cell lines. The result of CCK8 assay certified that the proliferation ability of both the A375 and A875 cells decreased upon exposure to the combined intervention group compared with the single treatment group, demonstrating that si-CNTFR can strengthen the therapeutic potential of chemotherapy drugs. On the whole, the findings of pharmacology experiments further emphasized the pivotal function of si-CNTFR, as a novel supplement of chemotherapeutic drugs that could provide a valid therapeutic option for patients suffering from melanoma (Figures 12I,J).

## Discussion

Developing from transformed melanocytes, melanoma is the most invasive and deadly form of skin cancer (Eddy and Chen, 2020). Thus, early diagnosis of melanoma is crucial, *in situ*, since it can be cured at this stage. As innovative technologies in bioinformatics and sequencing are developed, increasing understanding of the etiology and progression have appeared for melanoma patients. Recently, there is a growing emphasis on the critical role of early diagnosis and immunotherapy for a good prognosis of melanoma patients. Nevertheless, most bioinformatics analyses focus on epigenetic or transcriptional regulation without adequately examining the critical biological processes involved in melanoma pathogenesis and progression. As a result, existing prediction models are not fully reflective of the features of melanoma. Increasing evidence confirms the critical role of aging in tumor immunity, particularly immune evasion strategies, but the combined mechanisms of aging and immune in the prognosis of patients with melanoma are still not fully elucidated. Concurrently, immunotherapy has shifted from cytokine-based to antibody-mediated blocking of checkpoints such as CTLA-4 and PD-1. With particular attention to the immune checkpoint blocking exploited by melanomas, to circumvent host immune surveillance and assess clinical prognosis.

In this study, based on transcriptome sequencing data and respective clinical information in TCGA databases, we constructed a CIAI by combining ARGs and IRGs. It has been demonstrated that CIAI is capable of displaying an effective level of predictive performance in different datasets. Therefore, it can serve as an independent prognostic factor for patients with melanoma. Accordingly, CIAI’ capability to predict the prognosis of melanoma with considerable accuracy may be carried out in clinical practice. Additionally, our result clearly demonstrated that different CIAI scores have striking differences in clinical characteristics. Compared with a low-CIAI group, the high-CIAI group had a shorter survival time in both OS, DSS, and PFI, indicating that the CIAI score was positively correlated with poor prognosis.

Numerous risk models have been constructed to assess clinical outcomes in melanoma patients using transcriptome sequencing data from TCGA databases. Researchers at NIU et al. constructed a novel pyroptosis-related signature to predict prognosis in melanoma patients, and low-risk patients responded better to immune checkpoint inhibitors (Niu et al., 2022). Using autophagy-associated genes, Deng et al. (2021) established a promising nomogram for melanoma prognosis that outperformed traditional TNM staging. The ferroptosis-related gene signature constructed by Xu et al. (2021) can predict the prognosis of melanoma patients and have a potential utility in guiding targeted therapy. Analogously, a signature consisting of four pyroptosis-associated genes will provide a promising approach to predicting survival and prognosis of melanoma patients and facilitate the personalization of treatment (Wu et al., 2021). In the study of Tian et al. (2021), a robust 7 RNA binding protein signature was validated as a potential biomarker for cutaneous melanoma patients’ prognosis and immunotherapy response, contributing to advances in immunotherapy strategies (Tian et al., 2021). Nonetheless, these studies failed to adequately take into account the essential biological processes involved in tumor progression, especially aging and the immune microenvironment. Entertainingly, the CIAI model had the highest C-index among the above 5 signatures, indicating improved performance and reflecting cancer’s intrinsic characteristics. Our research was the first to construct a prognostic signature for two pivotal tumor advancing processes, aging and immunity, which can represent both the aging characteristics and immune status of melanoma. Importantly, the ROC curve and decision curve analysis (DCA) illustrated that the prospective nomogram was superior to the conventional TNM staging system in predicting melanoma outcome.

The CIAI we constructed consists of 7 genes, FOXM1, TP63, ARNTL, KIR2DL4, CCL8, SEMA6A, and CNTFR. Among these, FOXM1, TP63, and ARNTL were all related to aging, while KIR2DL4, CCL8, SEMA6A, and CNTFR have pertained to immune responses. To construct distinguished and more rational predictive models, we have adequately investigated and compared the existing generally recognized modeling strategy. For instance, based on Lasso-penalized and multivariate Cox regression analysis, Liu et al. established an immune-related genes prognostic signature to stratify the epithelial ovarian cancer patients and explore tumor immune microenvironment (Liu et al., 2020). Analogously, an excellent signature based on six pyroptosis-related lncRNAs was established using multiple Cox regression analysis for predicting prognoses and immune responses in uterine corpus endometrial carcinoma patients (Liu et al., 2022). Besides, an admirable and universal prognostic signature computed with principal component analysis algorithm was performed to quantify m1A modification pattern and assess immunological characteristics in ovarian cancer (Liu et al., 2021). After comprehensively considering the AUC values of the models constructed by diverse algorithms, in this study, the LASSO regression analysis and stepwise multivariate Cox regression analysis were applied to establish the CIAI signature. The study by SIU et al. found that radiation combined with FOXM1 inhibition was a potent inducer of cell death and suppressor of migration in metastatic melanoma cells (Lee et al., 2018). A previous study by Bergamasch et al. has shown that TP63 inhibits p53-induced apoptosis in melanoma cells following the application of genotoxic drugs, which may serve as a strategy to overcome resistance (Patel et al., 2020). The Antineoplastic effect of L-theanine on melanoma cells was relevant to its ability to inhibit proliferation and migration and promote apoptosis, depending on the timing of the clock gene ARNTL (Zhang et al., 2022). In the B16 melanoma metastasis model, MCP2/CCL8 exhibited direct repression of tumor cell proliferation or tumor cell invasion into surrounding normal tissue, confirming that MCP2/CCL8 possesses potential anti-tumor effect for tumor metastasis (Hiwatashi et al., 2011). SEMA6A belongs to the semaphorin family, and it interacts with plexins to regulate the actin cytoskeleton and cell proliferation. In BRAFV600E melanoma, SEMA6A was preferentially expressed by western blot and then silencing of SEMA6A induced cell death (Loria et al., 2015). Notably, the CNTFR was a promising therapeutic candidate for the treatment of a variety of tumors such as lung, colon, and pancreatic cancers and may portend specific biomarkers of increasing sensitivity. Interestedly, the CLCF1–CNTFR axis played an important pro-oncogenic role in lung cancer, and its inhibition exhibited significant therapeutic effects (Kim et al., 2019). Unfortunately, the role of CNTFR in melanoma in modulating tumor growth and sensitivity to immunotherapy have obtained much less attention. In this study, we presented the first evidence on CNTFR as a tumor progression-promoting gene and its prognostic value for melanoma. As a consequence of the molecular biology experiments, we have found that inhibiting CNTFR activity exerted a reverse regulatory role in the proliferation and migration of melanoma cells, but facilitated the expression of immune checkpoints (PD-1, PD-L1, and CTLA4).

Noteworthily, immunotherapy blocking PD-1, PD-L1, and CTLA4 is extensively employed for the treatment of various cancers, especially melanoma. Existing studies conducted that the unresponsiveness of PD-1, PD-L1, and CTLA4 contributed to the defective development of cytotoxic memory T cells and the depletion of CD8^+^ T cells. Patients with high PD-1, PD-L1, and CTLA4 checkpoint expression are normally more susceptible to immunosuppressive therapy and obtain a better clinical prognosis. Moreover, considering the mechanisms of resistance, as well as the complexity of immunotherapy biomarkers, we constructed a prognostic signature to assess the clinical benefits and limitations of immune checkpoint blocking in melanoma patients. In the research, we found that the expression of PD-1, PD-L1, and CTLA-4 was observed to be elevated in the low CIAI score group and predicted a greater survival prognosis. Simultaneously, in the TCGA cohort, the low CIAI score group may benefit more from the responsiveness of anti-PD1 therapy and classical chemotherapeutic agents. Combined analysis of CIAI score and immune checkpoint expression confirmed that the low CIAI score group with high PD1 expression possessed a longer survival time and exhibited immune activation, with the consistent conclusion of PD-L1 and CTLA-4. As is known, though ICI therapies have emerged as potent weapons against melanoma, heterogeneity of response as well as high expense limit their applications. Thus, treatment decisions for melanoma patients calls for more accurate references. According to this study, CIAI could serve as a precise prediction model for ICI therapy strategy making, along with existing predictors such as PD-L1/PD-1 and CTLA-4 expression level, TMB and melanoma subtypes. Also, CIAI could offer suggestion on the selection of chemotherapeutic and targeted drugs as CIAI score is correlated with the responsiveness of chemotherapy and target therapy. For instance, high CIAI score group would benefit more from AZ628, while low CIAI score group would take ABT.263 as a superior treatment option. Taken together, we hypothesize that the combination of CIAI score and ICI has great potential for clinical replication, and expect that our constructed signature may contribute to developing new combination treatment strategies and evaluating the sensitivity of classical chemotherapeutic agents.

Although our study suggested CIAI model can appear as an effective prognostic tool for application in melanoma patients, there are still some limitations to be addressed. First, future studies need to further clarify the stability and generality of the CIAI prognostic model using a large sample clinical cohort. Secondly, future studies should evaluate the therapeutic prospects and long-term outcomes of gene therapy combined with immune checkpoint inhibitor therapy for melanoma in animal experiments, and provide novel clinical auxiliary strategies for melanoma immunotherapy.

## Data Availability

The datasets presented in this study can be found in online repositories. The names of the repository/repositories and accession number(s) can be found in the article/Supplementary Material.

## Supplementary material

Supplementary Figure S1 The flowchart of this study.

Supplementary Figure S2 Kaplan-Meier survival curves of 7 CIAI genes in the melanoma patient.
