# Supplementary figures and images for: A combined aging and immune prognostic signature predict prognosis and responsiveness to immunotherapy in melanoma
Source: Front Pharmacol. 2022 Aug 11;13:943944. doi: 10.3389/fphar.2022.943944 (PMC9402914; doi:10.3389/fphar.2022.943944)

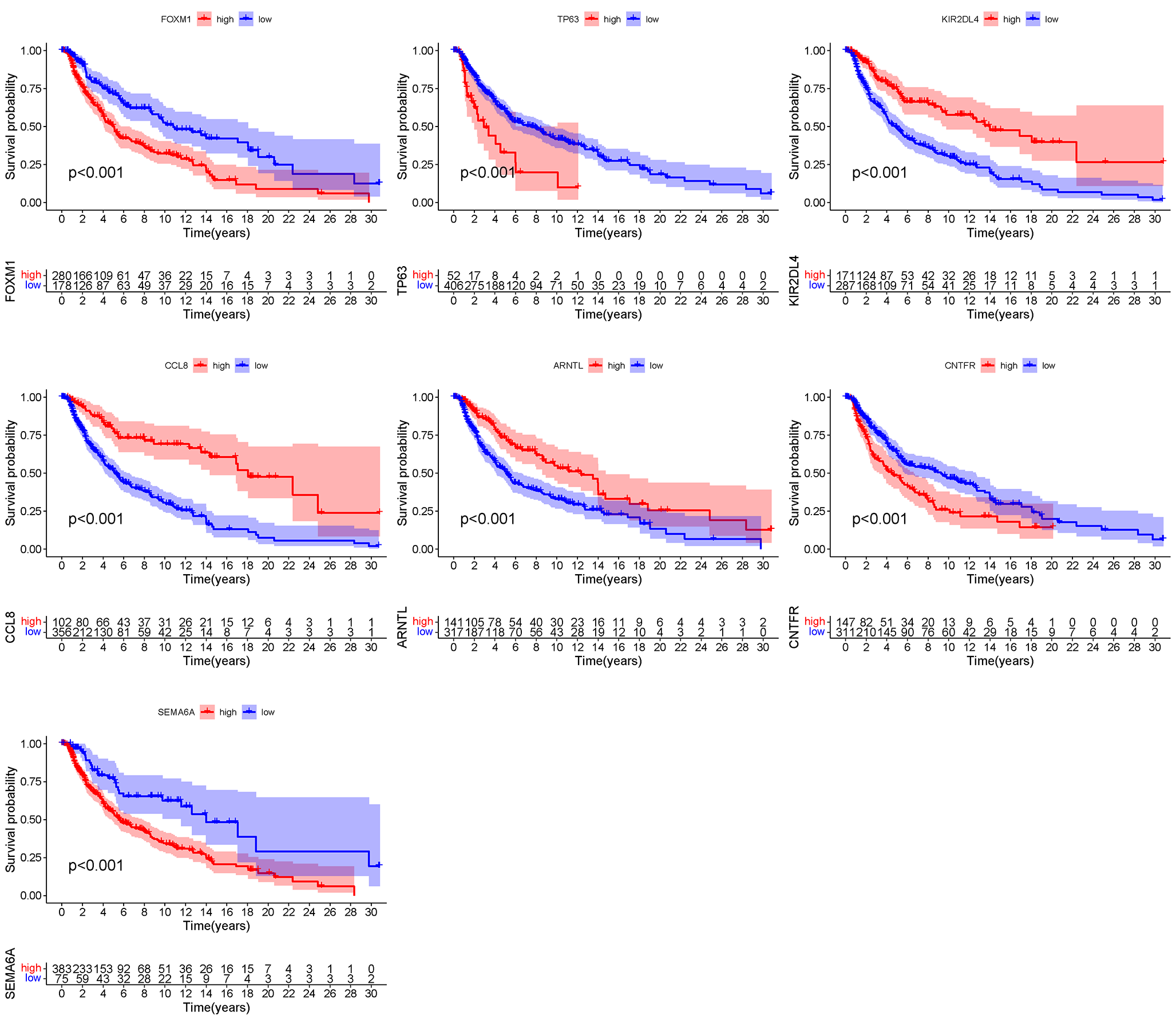

Supplement: Supplementary file 3 [file Image2.TIF]

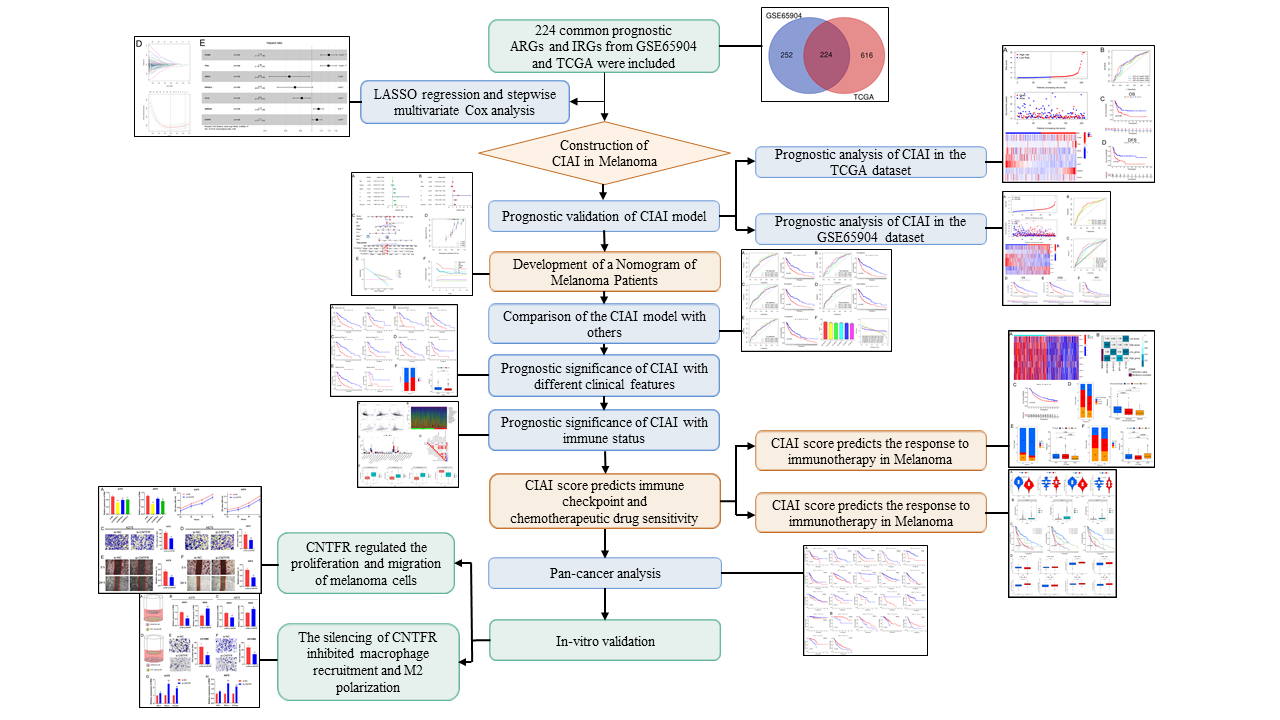

Supplement: Supplementary file 4 [file Image1.TIF]
